# The Complete Mitochondrial Genome of the Asiatic Cavity-Nesting Honeybee *Apis cerana* (Hymenoptera: Apidae)

**DOI:** 10.1371/journal.pone.0023008

**Published:** 2011-08-12

**Authors:** Hong-Wei Tan, Guo-Hua Liu, Xia Dong, Rui-Qing Lin, Hui-Qun Song, Si-Yang Huang, Zi-Guo Yuan, Guang-Hui Zhao, Xing-Quan Zhu

**Affiliations:** 1 State Key Laboratory of Veterinary Etiological Biology, Lanzhou Veterinary Research Institute, Chinese Academy of Agricultural Sciences, Lanzhou, Gansu Province, China; 2 College of Veterinary Medicine, Hunan Agricultural University, Changsha, Hunan Province, China; 3 Eastern Bee Research Institute, Yunnan Agricultural University, Kunming, Yunnan Province, China; 4 College of Veterinary Medicine, South China Agricultural University, Guangzhou, Guangdong Province, China; 5 College of Veterinary Medicine, Northwest A & F University, Yangling, Shaanxi Province, China; 6 Animal Husbandry Technology Promotion Station in Chongqing, Chongqing, China; 7 College of Animal Science and Veterinary Medicine, Heilongjiang Bayi Agricultural University, Daqing, Heilongjiang Province, China; Ghent University, Belgium

## Abstract

In the present study, we determined the complete mitochondrial DNA (mtDNA) sequence of *Apis cerana*, the Asiatic cavity-nesting honeybee. We present here an analysis of features of its gene content and genome organization in comparison with *Apis mellifera* to assess the variation within the genus *Apis* and among main groups of Hymenoptera. The size of the entire mt genome of *A. cerana* is 15,895 bp, containing 2 ribosomal RNA genes, 13 protein-coding genes, 22 transfer RNA (tRNA) genes and one control region. These genes are transcribed from both strands and have a nucleotide composition high in A and T. The contents of A+T of the complete genomes are 83.96% for *A. cerana*. The AT bias had a significant effect on both the codon usage pattern and amino acid composition of proteins. There are a total of 3672 codons in all 13 protein-coding genes, excluding termination codons. The most frequently used amino acid is Leu (15.52%), followed by Ile (12.85%), Phe (10.10%), Ser (9.15%) and Met (8.96%). Intergenic regions in the mt genome of *A. cerana* are 705 bp in total. The order and orientation of the gene arrangement pattern is identical to that of *A. mellifera*, except for the position of the *tRNA-*Ser^(AGN)^ gene. Phylogenetic analyses using concatenated amino acid sequences of 13 protein-coding genes, with three different computational algorithms (NJ, MP and ML), all revealed two distinct groups with high statistical support, indicating that *A. cerana* and *A. mellifera* are two separate species, consistent with results of previous morphological and molecular studies. The complete mtDNA sequence of *A. cerana* provides additional genetic markers for studying population genetics, systematics and phylogeographics of honeybees.

## Introduction

The Asiatic honeybee, *Apis cerana* Fabricius (Hymenoptera: Apidae), is an important honeybee species in Asian countries. For a long time *A. cerana* was considered a sub-species of *A. mellifera*. However, on the basis of several genetic, morphological, and behavioural characteristics, it has been established that *A. cerana* and *A. mellifera* are two completely isolated species, and they were considered most recently derived and sister taxa [1]–[4]. Honeybee has been the object of considerable scientific curiosity and has served as the classical model organism in diverse fields such as navigation, face recognition and sensory biology for almost a century [5]–[7]. Honeybee is regarded as the premier pollinator of major fruit crops. It has a long history of association with mankind because of its cavity-nesting lifestyle and it is used for producing honey, bee pollen, wax, and royal jelly [8].

Most metazoan species possess a compact, circular mitochondrial (mt) genome, which varies in size from 14 to 19 kb that contain 36–37 genes, including 12–13 protein-coding genes, two ribosomal RNAs (rRNA) genes and 22 transfer RNAs (tRNA) genes necessary for translation of the proteins encoded by the mtDNA [9]–[11]. mtDNA has been extensively used for studying phylogenetic and evolutionary relationships among animal species, due to its maternal inheritance, rapid evolutionary rate, and lack of genetic recombination [12]–[16]. mtDNA has proved to be an important tool in intra-specific and inter-specific phylogenetic studies of honeybees [17]–[21], and one region of the *Apis* mt genome, a non-coding sequence located between cytochrome oxidase I (*cox*1) and cytochrome oxidase II (*cox*2) has proven to be particularly informative for intra-specific studies [22], [23].

The Hymenoptera are one of the largest insect orders belonging to hexapods, comprising over 100,000 described species [24]. In 1993, Crozier reported the complete sequence of the *A. mellifera* mt genome, this was the first identified mt genome of Hymenopteran [25]. Until recently, only 9 complete and 18 nearly complete mt genome sequences have been determined in Hymenoptera [25]–[35] (Table 1), and in the genus *Apis*, only the *A. mellifera* mt genome has been determined. The lack of knowledge of mt genomics for Hymenoptera is a major limitation for population genetic and phylogenetic studies of the Hymenoptera including the species in the Apidae.

**Table 1:** Mitochondrial genome sequences of Hymenoptera sequenced completely or nearly completely prior to the present study and used for phylogenetic analysis.

Family	Taxon	Accession number	Reference
Apidae	*Apis mellifera*	L06178	Crozier and Crozier 1993
	*Bombus ignitus*	DQ870926	Cha et al. 2007
	*Bombus hypocrita*	EU401918	Unpublished
	*Melipona bicolor*	AF466146	Silvestre et al. 2008
Chrysididae	*Primeuchroeus* spp	AH015389	Castro et al. 2006
Pergidae	*Perga condei*	AY787816	Castro and Dowton 2005
Vanhorniidae	*Vanhornia eucnemidarum*	DQ302100	Castro et al. 2006
Vespidae	*Abispa ephippium*	EU302588	Cameron et al. 2008
	*Polistes humilis synoecus*	EU024653	Cameron et al. 2008
Pteromalidae	*Nasonia vitripennis* AsymC	EU746609	Oliveira et al. 2008
	*Nasonia vitripennis* HiCD12	EU746610	Oliveira et al. 2008
	*Nasonia giraulti* RV2	EU746611	Oliveira et al. 2008
	*Nasonia longicornis* IV7	EU746612	Oliveira et al. 2008
Cephidae	*Cephus cinctus*	FJ478173	Dowton et al. 2009
Orussidae	*Orussus occidentalis*	FJ478174	Dowton et al. 2009
Ichneumonidae	*Venturia canescens*	FJ478176	Dowton et al. 2009
	*Enicospilus* sp	FJ478177	Dowton et al. 2009
	*Diadegma semiclausum*	EU871947	Wei et al. 2009
Evaniidae	*Evania appendigaster*	FJ593187	Wei et al. 2009
Stephanidae	*Schlettererius cinctipes*	FJ478175	Dowton et al. 2009
Braconidae	*Cotesia vestalis*	FJ154897	Wei et al. 2010
	*Spathius agrili*	FJ387020	Wei et al. 2010
	*Phanerotoma flava*	GU097654	Wei et al. 2010
	*Diachasmimorpha longicaudata*	GU097655	Wei et al. 2010
	*Macrocentrus camphoraphilus*	GU097656	Wei et al. 2010
	*Meteorus pulchricornis*	GU097657	Wei et al. 2010
	*Aphidius gifuensis*	GU097658	Wei et al. 2010

In the present study, we determined the complete nucleotide sequence of the *A. cerana* mt genome, we performed phylogenetic analyses using selected Hymenoptera species. The new sequence may provide useful information on both genomics and the evolution of Hymenoptera, because there are only a few complete (or nearly complete) mtDNA sequences available from these animals.

## Results and Discussion

### General features of the mt genome of *A. cerana*

**Figure 1:**
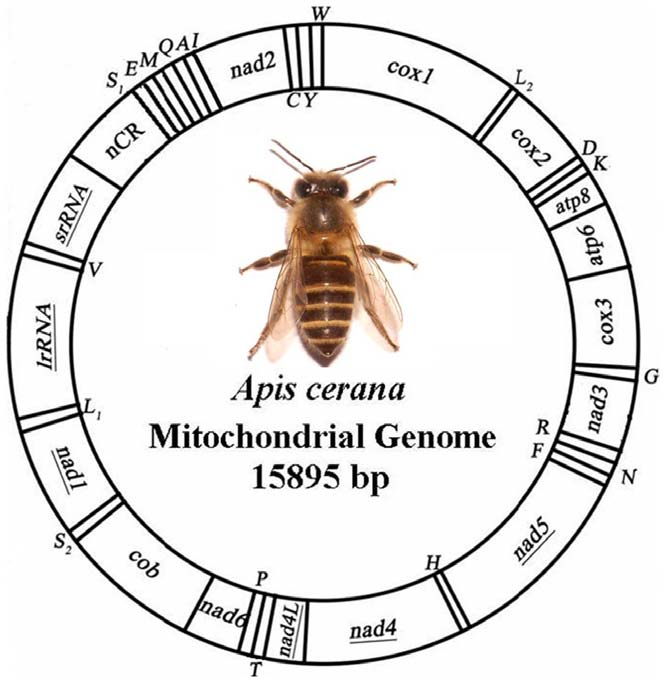
The mitochondrial genome of *Apis cerana*. Protein-coding genes are transcribed in a clockwise direction, except for those underlined. The two ribosomal RNA genes were encoded by the L strand. Transfer RNA genes encoded by H and L strands are shown outside and inside the circular gene map, respectively. Transfer RNA genes are designated by single-letter amino acid codes, except those encoding leucine and serine, which are labeled L_1,_ L_2,_ S_1,_ S_2_, representing *tRNA-*Leu^(CUN)^, *tRNA-*Leu^(UUR)^, *tRNA*-Ser^(AGN)^ and *tRNA*-Ser^(UCN)^, respectively.

The length of the complete mt genome of *A. cerana* was 15,895 bp (Figure 1), and the mtDNA sequence was deposited in the GenBank under the accession number GQ162109. The mt genome of *A. cerana* contains 13 protein-coding genes (*cox*1-3, *nad*1-6, *nad*4L, *atp*6, *atp*8 and *co*b), a small subunit ribosomal RNA gene (*rrn*S), a large subunit ribosomal RNA gene (*rrn*L), and 22 transfer RNA genes (Table 2). The *A. cerana* mt genome shows a novel arrangement within Hymenoptera compared with the ancestral pancrustacean mt genome organization [36]. All rearranged genes are tRNAs, tRNA gene rearrangements can be classified as translocations, local inversions, or shuffling. A translocation is a movement of a gene to another position across a protein-coding gene. A rearrangement is classified as an inversion when the tRNA is found on the opposite strand and as shuffling when the tRNA gene is on the same mt stand but in a different position compared with the ancestral organization (without movement across a protein-coding gene) [37].

**Table 2:** Positions and nucleotide sequence lengths of mitochondrial genomes of *Apis cerana*, and start and stop codons for protein-coding genes as well as their tRNA gene anticodons (starting from *tRNA*-S_1_).

Gene/Region	Position		Size (bp)	Strand	Anticodon	Codon		Intergenic nucleotides^a^
	Form	To				Start	Stop	
*tRNA*-Ser^(AGN)^ (S_1_)	1	60	60	H	TCT			3
*tRNA*-Glu (E)	64	129	66	H	TTC			34
*tRNA*-Met (M)	164	229	66	H	CAT			231
*tRNA*-Gln (Q)	461	522	62	H	TTG			0
*tRNA*-Ala (A)	523	588	66	H	TGC			18
*tRNA*-Ile (I)	607	672	66	H	GAT			0
*nad*2	673	1668	996	H		ATT	TAA	−1
*tRNA*-Cys (C)	1668	1733	66	L	GCA			5
*tRNA*-Tyr (Y)	1739	1807	69	L	GTA			16
*tRNA*-Trp (W)	1824	1892	69	H	TCA			0
*cox*1	1893	3458	1566	H		ATT	TAA	−5
*tRNA*-Leu^(UUR)^ (L_2_)	3454	3523	70	H	TAA			89
*cox*2	3613	4293	681	H		ATT	TAA	−2
*tRNA*-Asp (D)	4292	4359	68	H	GTC			6
*tRNA*-Lys (K)	4366	4437	72	H	TTT			6
*atp*8	4444	4605	162	H		ATC	TAA	−19
*atp*6	4587	5264	678	H		ATG	TAA	17
*cox*3	5282	6061	780	H		ATG	TAA	66
*tRNA*-Gly (G)	6128	6194	67	H	TCC			0
*nad*3	6195	6548	354	H		ATT	TAA	19
*tRNA*-Arg (R)	6568	6633	66	L	TCG			19
*tRNA*-Asn (N)	6653	6720	68	H	GTT			18
*tRNA*-Phe (F)	6739	6809	71	L	GAA			6
*nad*5	6816	8486	1671	L		ATT	TAA	0
*tRNA*-His (H)	8487	8552	66	L	GTG			17
*nad*4	8570	9898	1329	L		ATA	TAA	0
*nad*4L	9899	10162	264	L		ATT	TAA	23
*tRNA*-Thr (T)	10186	10252	67	H	TGT			15
*tRNA*-Pro (P)	10268	10345	78	L	TGG			50
*nad*6	10396	10905	510	H		ATT	TAA	12
*co*b	10918	12066	1149	H		ATG	TAA	23
*tRNA*-Ser^(UCN)^ (S_2_)	12090	12156	67	H	TGA			12
*nad*1	12169	13083	915	L		ATT	TAA	0
*tRNA-*Leu^(CUN)^ (L_1_)	13084	13152	69	L	TAG			0
*rrn*L	13153	14480	1328	L				0
*tRNA*-Va*l* (V)	14481	14547	67	L	TAC			0
*rrn*S	14548	15333	786	L				0
AT-rich region (nCR)	15334	15895	562					0

a indicates gap nucleotides (positive value) or overlapped nucleotides (negative value) between two adjacent genes.

**Figure 2:**
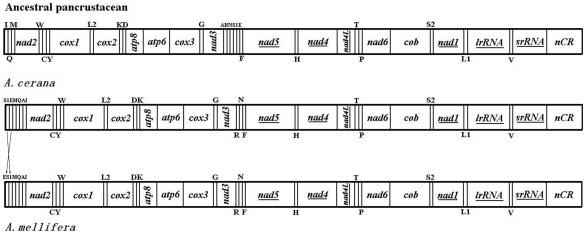
Comparison of the mitochondrial gene arrangement among *A. mellifera*, *A. cerana* and ancestral pancrustacean. tRNA genes are labeled by one-letter symbol on the bar when transcription starts from right to left direction, or under the bar when transcription starts from left to right direction. Underlined PCGs or rRNA genes are transcribed from right to left and PCGs that are not underlined are transcribed from left to right.

As shown in Figure 2, we identified eight rearrangements shared between *A. cerana* and *A. mellifera*. *tRNA-*Ala, *tRNA-*Ser^(AGN)^ and *tRNA-*Glu move to *tRNA-*Ile—*tRNA-*Gln—*tRNA-*Met cluster, and *tRNA-*Ala changes position relative to *tRNA-*Ser^(AGN)^ and *tRNA-*Glu. *tRNA-*Trp moves across two tRNAs (*tRNA-*Cys and *tRNA-*Tyr) boundaries. The order of *tRNA-*Ile—*tRNA-*Gln—*tRNA-*Met becomes *tRNA-*Met—*tRNA-*Gln—*tRNA-*Ile, and two of tRNAs (*tRNA-*Gln and *tRNA-*Arg) arrangements are inversions. Finally, the *tRNA-*Lys and *tRNA-*Asp swap positions. The orientation and gene order of the *A. cerana* mt genome were identical to that of *A. mellifera* except for the *tRNA-*Ser^(AGN)^. In *A. cerana*, *tRNA-*Ser^(AGN)^ gene was only translocated from *nad*3-*nad*5 junction to the junction of *nad*2 and AT-rich region compared with the ancestral pancrustacean mt genome organization. In *A. mellifera*, *tRNA-*Ser^(AGN)^ gene was not only translocated but also shuffled, which swapped positions with *tRNA*-Glu gene. The duplication/random loss model, and the intramitochondrial genome recombination and duplication/nonrandom loss model are possible mechanisms to explain translocation and shuffling. Of these, intramitochondrial genome recombination is presumed to be more common [37].

The genes *atp*6, *atp*8, *cox*1, *cox*2, *cox*3, *co*b, *nad*2, *nad*3 and *nad*6 are transcribed from one strand, while *nad*1, *nad*4, *nad*4L, and *nad*5 are transcribed from the other strand. The nucleotide compositions of the entire mtDNA sequences for *A. cerana* are biased toward A and T, with T being the most favored nucleotide and G the least favored, in accordance with the mt genome of *A. mellifera*. The content of A+T is 83.9% for *A. cerana* (42.3% A, 41.6% T, 6.3% G and 9.8% C) (Table 3), 84.9% for *A. mellifera* (43.2% A, 41.7% T, 5.5% G and 9.6% C), respectively. The bias of the base composition of an individual strand can be described by skewness [38], which is calculated as (A%−T%)/(A%+T%) and (G%−C%)/(C%+G%), respectively. AT-skews and GC-skews of each of the 13 protein-coding genes and the 2 ribosomal RNA genes and the whole mt genome were calculated for *A. cerana* and *A. mellifera* (Table 3). In *A. cerana*, except for the *atp*8 gene which has a relatively weak skew of A *vs* T (AT skew = 0.092), other twelve PCGs have a skew of T *vs* A (AT skew between −0.002 and −0.209). The PCGs of H-strand have a strong skew of C *vs* G (GC skew between −0.098 and −0.309), and the *nad*1, *nad*4, *nad*4L and *nad*5 genes, which coded on the L-strand have a strong skew of G *vs* C (GC skew between 0.252 and 0.470). In the two rRNA genes, the GC skew displayed the same pattern (GC skew = 0.352 and 0.312 for the *rrn*S and *rrn*L genes, respectively). The AT skew displayed an opposite pattern (AT skew = 0.076 and −0.029 for *rrn*S and *rrn*L genes, respectively). By comparing the skew values of 13 protein-coding genes and two rRNA genes between *A. cerana* and *A. mellifera*, we found that GC skews of different genes coded on the same strand are all positive or negative, whereas the AT skews of different genes coded on the same strand are either positive or negative. This is congruent with the pattern of skew values in other insects [39]. Wei et al. (2010) found that the criterion for detecting reversal of strand asymmetry on mt genomes should be the sign of GC skew values, not the AT skew values.

**Table 3:** Nucleotide composition and skews of *Apis cerana* mitochondrial protein-coding and ribosomal RNA genes and comparison with *Apis mellifera*.

Gene	Nucleotide frequency				%A+T	AT-skew	GC-skew	AT-skew	GC-skew
	A	G	T	C				*A. mellifera*	
*atp*6	0.366	0.056	0.472	0.106	83.8	−0.127	−0.309	−0.099	−0.269
*atp*8	0.475	0.043	0.395	0.086	87.0	0.092	−0.333	0.042	−0.413
*cox*1	0.347	0.110	0.413	0.130	76.0	−0.086	−0.085	−0.087	−0.093
*cox*2	0.385	0.087	0.404	0.125	78.9	−0.024	−0.181	−0.029	−0.167
*cox*3	0.360	0.089	0.444	0.108	80.4	−0.104	−0.098	−0.113	−0.188
*co*b	0.368	0.084	0.442	0.105	81.0	−0.091	−0.110	−0.087	−0.148
*nad*1	0.355	0.109	0.480	0.056	83.5	−0.149	0.325	−0.184	0.359
*nad*2	0.395	0.051	0.469	0.085	86.4	−0.086	−0.250	−0.091	−0.304
*nad*3	0.373	0.051	0.475	0.102	84.8	−0.120	−0.334	−0.105	−0.375
*nad*4	0.360	0.093	0.491	0.056	85.1	−0.153	0.252	−0.161	0.337
*nad*4L	0.345	0.095	0.527	0.034	87.2	−0.209	0.470	−0.234	0.676
*nad*5	0.379	0.095	0.470	0.056	84.9	−0.107	0.257	−0.115	0.297
*nad*6	0.431	0.051	0.433	0.084	86.4	−0.002	−0.246	−0.014	−0.545
*rrn*S	0.439	0.125	0.377	0.060	81.6	0.076	0.352	0.028	0.370
*rrnL*	0.404	0.111	0.428	0.058	83.1	−0.029	0.312	−0.043	0.375
Total	0.423	0.063	0.416	0.098	83.9	0.008	−0.217	0.018	−0.272

AT skew = (A%−T%)/(A%+T%); GC skew = (G%−C%)/( G%+C%).

The *A. cerana* mt genes overlap a total of 27 bp in 4 locations ranging from 1 to 19 bp (Table 2). Gene overlaps on the same strand in the mt genome of *A. cerana* can be founded in three regions. The longest is a 19 bp overlap between *atp*8 and *atp*6. The second case is a 5 bp overlap between *cox*1 and *tRNA*-Leu^(UUR)^. The third is 2 bp overlap occurred between *cox*2 and *tRNA*-Asp. Overlaps of genes coded by the different strands also can be found in the mt genome of *A. cerana*. one bp overlap exists between *nad*2 and *tRNA*-Cys. Interestingly, the *A. cerana* mt genome has the same overlapping nucleotides and locations between genes as those of *A. mellifera*, where genes also overlap a total of 27 bp in four locations [25].

Apart form the A+T-rich region, the *A. cerana* mt genes are separated by a total of 705 bp of intergenic spacer sequences, which are spread over 22 regions and range in size form 1 to 231 bp (Table 2). The longest intergenic region (231 bp) is located between *tRNA*-Met and *tRNA*-Gln genes. In *A. mellifera* mt genome, a total of 813 bp of intergenic spacer sequences are spread over 24 regions, ranging in size from 1 to 193 bp. The longest one is located between *tRNA*-Leu^(UUR)^ and *cox*2 [25]. This region has proven to be particularly informative for intraspecific studies, because the sequences do not appear to be subject to strong purifying selection and accumulate numerous base substitutions and insertion/deletions [21]. In comparison, *A. cerana* has only 89 bp intergenic spacer sequence in this region. Such short intergenic regions were also found in other metazoans [40]–[42]. Furthermore, previous studies have shown that the intergenic regions in the mt genomes of some metazoan contain signals for transcription initiation and replication [43], [44]. In the spacer region (*tRNA*-Ser^(UCR)^–*nad*1), we found a 6 bp motif (TACTTA), which is conserved between *A. cerana* and *A. mellifera*. This intergenic spacer region may correspond to the binding site of mtTERM, a transcription attenuation factor [45].

### Protein-coding genes and codon usage patterns

The boundaries between protein-coding genes in the mt genome of *A. cerana* were determined by aligning their sequences and by identifying translation initiation and termination codons with comparison to those of *A. mellifera*. The amino acid sequences inferred for *nad*3, *nad*4L, *cox*1 and *cox*3 genes are similar in length to those of *A. mellifera* (Table 4). The similarity of the amino acid sequences is 51.5%–97.5% between *A. cerana* and *A. mellifera* (Table 4). Based on similarity, *cox*1 is the most conserved protein-coding gene, while the *nad*6 is the least conserved. Genes in mt genome may have different evolutionary rates, which might be caused by different selection pressures or the restriction of gene function. That is what we found in honeybee that rates of different mitochondrial genes diverged differently.

**Table 4:** Protein-coding gene assignments and similarity between *A. cerana* and *A. mellifera*.

Protein-coding gene	Number of amino acids		Identity (%)
	*A. cerana*	*A. mellifera*	*A. cerana*/*A. mellifera*
*atp*8	53	52	73.1
*atp*6	225	226	89.4
*nad*1	304	305	88.2
*nad*2	331	333	70.1
*nad*3	117	117	82.1
*nad*4	439	447	82.7
*nad*4L	87	87	85.1
*nad*5	556	554	82.7
*nad*6	169	167	51.5
*cox*1	521	521	97.5
*cox*2	226	225	95.6
*cox*3	259	259	78.8
*co*b	382	383	91.9

The predicted translation initiation and termination codons for the protein-coding genes of *A. cerana* mt genome were compared with those of *A. mellifera*. All protein-coding sequences started with a typical ATN codon. The start codons inferred in the mt genome of *A. cerana* are ATC (one of 13 protein-coding genes), ATT (eight of 13 protein-coding genes), ATG (three of 13 protein-coding genes) and ATA (one of 13 protein-coding genes) as a translation initiation codon (Table 2). Furthermore, the anomalous initiation codon and incomplete stop codon frequently occur in protein-coding genes of most of the Hymenoptera mtDNA sequenced to date, such as the gene *cox*1 has been found to employ TTG as a start codon and *nad*4 has been found to employ TA as a stop codon in *Vanhornia eucnemidarum* [27]. The codon TGA, which is a termination codon in the universal code, codes for tryptophan in most mt genomes except those of higher plants [46]. All stop codons used TAA as a termination codon in the mt genome of *A. cerana*. This is presumably polyadenylation after transcription to complete the termination codon [47]. However, two of the 13 protein-coding genes (*cox*1, *cox*2) are predicted to employ T as an initiation codon in the mt genome of *A. mellifera* [25]. Furthermore, incomplete stop codon frequently occurs in protein-coding genes of most of the Hymenoptera mt DNA sequenced to date [27], [28], [32], [34].

The pattern of codon usage in the *A. cerana* mtDNA was also studied. Excluding the termination codons, a total of 3672 amino acids are encoded by the *A. cerana* mt genome, and a total of 3676 amino acids for *A. mellifera*. The most frequently used amino acids were Leu (15.52%), followed by Ile (12.85%), Phe (10.10%), Ser (9.15%) and Met (8.96%) (Table 5).

**Table 5:** Codon usage in 13 protein-coding genes of the *Apis cerana* mitochondrial genome.

Aa	Codon	N	RSCU	Aa	Codon	N	RSCU	Aa	Codon	N	RSCU	Aa	Codon	N	RSCU
Phe	UUU	336	1.81	Ser	UCU	55	1.31	Tyr	UAU	202	1.84	Cys	UGU	26	2.00
	UUC	35	0.19		UCC	9	0.21		UAC	17	0.16		UGC	0	0
Leu	UUA	483	5.08		UCA	167	3.98	End	UAA	0	0	Trp	UGA	84	2.00
	UUG	10	0.11		UCG	0	0		UAG	0	0		UGG	0	0
Leu	CUU	40	0.42	Pro	CCU	34	1.30	His	CAU	55	1.80	Arg	CGU	9	0.95
	CUC	1	0.01		CCC	4	0.15		CAC	6	0.20		CGC	1	0.11
	CUA	34	0.36		CCA	67	2.55	Gln	CAA	39	2.00		CGA	28	2.95
	CUG	2	0.02		CCG	0	0		CAG	0	0		CGG	0	0
lle	AUU	452	1.92	Thr	ACU	38	1.26	Asn	AAU	223	1.88	Ser	AGU	15	0.36
	AUC	20	0.08		ACC	1	0.03		AAC	14	0.12		AGC	0	0
Met	AUA	312	1.90		ACA	82	2.71	Lys	AAA	156	1.91		AGA	85	2.02
	AUG	17	0.10		ACG	0	0		AAG	7	0.09		AGG	5	0.12
Val	GUU	83	2.21	Ala	GCU	29	1.63	Asp	GAU	60	1.94	Gly	GGU	44	1.30
	GUC	7	0.19		GCC	4	0.23		GAC	2	0.06		GGC	3	0.09
	GUA	59	1.57		GCA	36	2.03	Glu	GAA	77	1.86		GGA	82	2.43
	GUG	1	0.03		GCG	2	0.11		GAG	6	0.14		GGG	6	0.18

A total of 3672 codons for *A. cerana* were analyzed, excluding the termination codons.

Aa = Amino acid; N = frequency of each codon; RSCU = relative synonymous codon usage.

### Transfer RNA genes

The 22 tRNA genes encoded in the mt genome of the *A. cerana* vary in length from 60 to 78 nucleotides with differences in stem and loop sizes of dihydrouridine (D) and TΨC loops. The order and orientation of the gene arrangement pattern are identical to that of *A. mellifera*, except for the position of the *tRNA-*Ser^(AGN)^ gene. All of the 22 tRNA genes have the typical cloverleaf structure, except for *tRNA*-Ser^(AGN)^ that lacks DHU arm (Figure S1). Their putative secondary structures (Figure S1) are similar to those of *A. mellifera* [25], indicating their similar functions. Among Hymenopteran, mismatched base pairs have been reported in mitochondrial tRNAs inferred for *A. mellifera*, *Melipona bicolor*, *Diadegma semiclausum*, *Bombus ignitus* and *Evania appendigaster* [25], [28], [31], [32], [34]. A total of 6 mismatched base pairs occur in tRNAs of *A. cerana*. Two tRNA genes, *tRNA*-Ala and *tRNA*-Gln, have a single G-T and T-T mismatches in the acceptor stem, respectively. *tRNA-*Val has a single T-T mismatch in the anticodon arm, and *tRNA*-Cys, *tRNA*-His and *tRNA*-Pro all have a single G-T mismatches in the DHU arm. Sequences of three tRNAs overlap with adjacent genes, *tRNA*-Cys overlaps with the adjacent gene *nad*2 on the opposite strand for 1 bp at its 3′-end. *tRNA*-Leu^(UUR)^ overlaps with the adjacent gene *cox*1 on the same strand for 5 bp at its 3′-end. *tRNA*-Asp overlaps with the adjacent gene *cox*2 on the same strand for 2 bp at its 3′-end. Finally, and most importantly, stem mismatches and sequence overlap are not uncommon for mt tRNAs of Hymenopteran, and are probably repaired by a post-transcriptional editing process [48], [49]. The 22 tRNA genes are located on both strands, 14 tRNAs are encoded on the H-strand and 8 on the L-strand (Table 2). In these tRNAs, there is a strict conservation of the sizes of the amino acid acceptor stem (13–15 bp) and the anticodon loop (7–9 bp). Their D-loops consist of 4–10 bp. The extra variable loops of 4–5 bp and the TΨC loops of 4–10 bp complete the cloverleaf structures. In most metazoan mitochondria, *tRNA*-Ser^(AGN)^ generally has an unpaired DHU arm, while *tRNA*-Ser^(UCN)^ has a standard cloverleaf structure [50], [51]. The *Apis tRNA*-Ser^(AGN)^ and *tRNA*-Ser^(UCN)^ structures conform to this interpretation.

### Ribosomal RNA genes

The *rrn*S and *rrn*L genes of *A. cerana* were identified by sequence comparison with those of *A. mellifera*. The *rrn*S is located between *tRNA*-Leu^(CUN)^ and *tRNA-*Val, and the *rrn*L is located between *tRNA*-Val and the A+T-rich region. The length of the *rrn*S and *rrn*L gene of *A. cerana* is 786 bp and 1328 bp, respectively, but relatively shorter than the genes in *A. mellifera* (827 bp and 1371 bp respectively) (Table 2). The A+T contents of the *rrn*S and *rrn*L for *A. cerana* are 81.6% and 83.1%, respectively (Table 3). Sequence identity in the *rrn*S and *rrn*L genes is 53.9% and 86.0% between *A. cerana* and *A. mellifera*, respectively.

### A+T-rich region

The A+T-rich region is believed to be involved in the regulation of transcription and control of DNA replication, characterized by five elements: (1) a polyT stretch at the 5′ end of the A+T-rich region, which may be involved in the control of transcription and/or replication initiation; (2) a [TA(A)]_n_-like stretch following the polyT stretch; (3) a stem and loop structure, which may be associated with the second strand-replication origin; (4) a TATA motif and a G (A)_n_T motif flanking the stem and loop structure, and (5) a G+A rich sequence downstream of the stem and loop structure [52]. The A+T-rich region is well known for the initiation of replication in both vertebrates and invertebrates, and the reduced G+C content is one of the most outstanding features of this region [9].

The largest non-coding region (562 bp) in the *A. cerana* mt genome is flanked by *rrn*S and *tRNA*-Ser^(AGN)^, it is highly enriched in AT (95.5%). In this region, we found some of the elements commonly present in most insects' A+T-rich region, such as PolyT stretch, [TA(A)]_n_-like stretch, stable stem-loop secondary structures (not shown), and TATA motif, which may function in the initiation of genome replication. Based on these features, it possibly functions as a control region. The AT-region of *Diadegma semiclausum* has the highest percentage of A+T content (96.4%) and *Orussus occidentalis* has the lowest A+T content (79.7%) among Hymenopteran sequenced to date (Table 6).

**Table 6:** Comparison of A+T content (%) of the AT region, protein-coding and rRNA genes of mitochondrial genomes of Hymenopteran.

Gene/Region	AE	AC	AM	BH	BI	CC	CV	DS	ESP	EA	MB	OO	PSP	PH	SA	VE
AT-region	89.9	95.9	96.0	/	96.0	88.3	/	96.4	88.1	85.6	/	79.7	78.9	/	/	/
*atp*8	86.1	87.0	89.3	92.6	92.6	82.8	93.0	89.5	91.7	69.1	91.1	82.4	86.5	93.7	90.1	82.7
*atp*6	80.4	83.8	84.7	85.0	85.4	77.3	86.4	84.1	84.6	73.9	86.6	74.8	77.6	83.1	84.3	76.3
*nad*1	80.6	83.5	83.0	82.7	84.7	79.8	85.3	85.2	82.3	74.6	86.4	75.0	75.4	84.7	79.0	79.0
*nad*2	80.9	86.4	86.5	89.0	89.8	85.8	91.2	89.2	88.9	80.5	91.2	75.3	80.4	89.9	88.8	83.7
*nad*3	80.5	84.8	86.4	88.3	90.0	78.3	88.7	85.0	87.3	74.1	89.0	75.0	78.7	84.5	83.5	79.9
*nad*4	82.1	85.1	86.5	85.5	85.9	81.3	87.5	85.8	86.0	77.8	89.2	77.0	77.4	84.1	83.7	80.1
*nad*4L	83.5	87.1	85.6	86.1	88.0	85.0	88.7	89.1	88.1	77.5	90.7	82.5	81.3	89.1	87.6	85.0
*nad*5	80.7	84.9	85.7	86.9	87.4	80.5	88.8	86.0	86.3	76.3	88.6	77.4	77.9	85.7	84.2	80.6
*nad*6	84.5	86.5	86.9	92.3	92.1	85.1	92.3	90.2	90.1	78.7	93.0	77.3	82.5	90.3	88.8	82.7
*rrn*S	80.4	81.6	81.4	85.4	86.6	80.5	91.2	88.5	89.4	75.8	82.8	81.1	77.1	84.0	89.3	80.8
*rrn*L	82.9	83.1	84.5	85.3	85.5	84.6	89.7	88.0	87.2	79.7	86.8	80.1	79.4	87.3	88.8	82.9
*cox*1	72.0	76.0	75.9	76.4	77.6	72.7	77.2	76.1	77.6	68.8	79.2	75.3	70.3	75.3	75.8	71.7
*cox*2	75.5	78.9	80.5	81.0	82.0	74.9	83.3	81.3	82.6	72.0	83.0	72.1	73.7	80.5	80.4	74.0
*cox*3	75.4	80.4	83.0	82.6	84.8	75.6	83.6	80.2	80.2	73.9	84.7	72.1	72.3	80.5	77.2	77.0
*co*b	75.6	81.0	80.6	81.0	82.8	77.0	82.3	80.1	81.0	73.3	82.6	71.1	73.0	81.2	78.9	75.7
EmtG	80.6	84.0	84.9	85.3	86.8	82.0	87.2	87.4	85.2	77.8	86.7	76.2	77.0	84.7	84.0	80.1

AE: *Abispa ephippium*, AC: *Apis cerana*, AM: *Apis mellifera*, BH: *Bombus hypocrita*, BI: *Bombus ignitus*, CC: *Cephus cinctus*, CV: *Cotesia vestalis*, DS: *Diadegma semiclausum*, ESP: *Enicospilus sp*, EA: *Evania appendigaster*, MB: *Melipona bicolor*, OO: *Orussus occidentalis*, PSP: *Philaenus spumarius*, PH: *Polistes humilis*, SA: *Spathius agrili*, VE: *Vanhornia eucnemidarum*, EmtG: entire mitochondrial genome.

### Phylogenetic analyses

**Figure 3:**
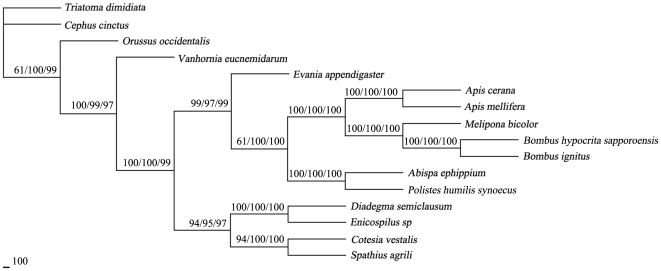
Inferred phylogenetic relationship among the Hymenoptera species. The analyses were conducted using maximum parsimony (MP), maximum likelihood (ML) and neighbour joining (NJ) of amino acid sequences of 13 protein-coding genes, using *Triatoma dimidiata* as outgroup. The numbers along branches indicate bootstrap values resulting from different analyses in the order: MP/ML/NJ.

Many systematic and population genetic studies have been based on genetic markers in the mt genomes at both the nucleotide and amino acid levels [53], [54]. So far, three mt genes (*rrn*L, *cox*2, *nad*2) and two nuclear gene (EF1-α intron and *itpr*) have been used for phylogenetic study of members within the genus *Apis*, yielding discrepant results [3], [4]. Usage of complete mt sequences for phylogenetic analyses would be more reliable, but so far only two complete mt genomes (*A. cerana* and *A. mellifera*) are available within the genus *Apis*. To better understand the evolution of genome-level features in *A. cerana*, phylogenetic relationships among representative members of the Hymenoptera were inferred from concatenated amino acid sequences of the 13 mt protein-coding genes. With addition of the *A. cerana* mt genome sequences, there are now 28 Hymenopteran sequences available for analysis, and only 16 of these have the complete sequences of the 13 mt protein-coding genes. The final alignment of the amino acid sequences of 13 protein genes for the 16 taxa were subjected to the ML, MP and NJ analyses. The results revealed that *A. cerana* and *A. mellifera* were closely related with high statistical support, indicating that *A. cerana* and *A. mellifera* are sister relationship (Figure 3), supporting the taxonomic classification by the analyses of morphological and molecular data. Three major clades were recovered within Apidae: Apini (*A. cerana* and *A. mellifera*), Meliponini (*M. bicolor*), and Bombini (*B. ignitus*+*B. hypocrita sapporoensis*) that were strongly supported in all (ML, MP and NJ) analyses. These results were congruent with previous studies [33], [35]. Our data provide a robust support for the relationship among Hymenopteran.

In conclusion, the mt genome of *A. cerana* exhibits almost the same characteristics with that of *A. mellifera*. Phylogenetic analyses indicated that *A. cerana* and *A. mellifera* are two separate species, supporting the taxonomic classification by genetic relatedness and phylogeny. The analyses of *A. cerana* mt genome have added to our knowledge on mt genomes of Hymenoptera, and provided additional genetic markers for studying population genetics, systematics and phylogeographics of honeybees.

## Materials and Methods

### Sample origin and DNA extraction

One *A. cerana* female adult was collected from the Mengla county, Yunnan province, southeastern China, and was preserved in 75% ethanol and stored at 4°C until used for DNA extraction. Total genomic DNA was extracted from its thoracic muscle tissue by treatment with sodium dodecyl sulphate/proteinase K (Merck), followed by purification using Wizard™ DNA Clean-Up System (Promega) and then eluted into 60 µl H_2_O according to the manufacturer's recommendations. DNA samples were stored at −20°C until further use.

### Amplification and sequencing of partial *cox*1, *nad*4 and *rrn*L

Partial fragments of the *cox*1, *nad*4 and *rrn*L mtDNA genes were amplified using three sets of primers (Table 7), designed by the authors according to homological mtDNA fragments of other Hymenopteran deposited in GenBank. PCR reactions were carried out in a 25 µl reaction volume consisting of 14.25 µl sterile deionized water, 2.5 µl 10×PCR Buffer (Mg^2+^ free), 4.0 µl MgCl_2_ (25 mM), 2.0 µl dNTPs (2.5 mM each), 0.25 µl each primer (50 pmol/µl), 0.25 µl ExTaq DNA polymerase (5 U/µl, Takara) and 1.5 µl DNA template (40 ng/µl) with the following conditions: after an initial denaturation at 94°C for 5 min, then 94°C for 30 s (denaturation), 45–50°C for 30 s (annealing), 72°C for 30 s (extension) for 35 cycles, followed by 72°C for 5 min (final extension). Each amplicon (5 µL) was examined by agarose gel electrophoresis to validate amplification efficiency. Then, the partial *cox*1, *nad*4 and *rrn*L amplicons were sent to TaKaRa Company (Dalian, China) for sequencing from both directions by using primers used in the PCR amplifications.

**Table 7:** Sequences of primers used to amplify PCR fragments from the *Apis cerana* mitochondrial genome.

Primers	Sequence (5′-3′)	Source	Estimated size of PCR products
AC-*cox*1F	ATTAGATTCTGATTGCTTCCT	This study	680 bp
AC-*cox*1R	TATGTTGCTAATCATCTAAAT	This study	
AC-*nad*4F	ATTAATCGTCTATTAGTTTG	This study	370 bp
AC-*nad*4R	TAAAAGCTCATGTTGAAGC	This study	
AC-*rrn*LF	TGAACTCAAATCATGTAAGAT	This study	450 bp
AC-*rrn*LR	ACTGTACAAAGGTAGCATAAT	This study	
AC-L*cox*1F	AGAAATTTATTTTATCCAAGACCAGGAACAG	This study	7.1 kb
AC-L*nad*4R	TGAAATTAGGAGGTTATGGGATATTACGTT	This study	
AC-L*nad*4F	AACATTAACTAAAAAAAATAAACCTGAAGAT	This study	4.8 kb
AC-L*rrn*LR	CCATGAAATAAATTTAAATAGCTGCAGTA	This study	
AC-L*rrn*LF	TGCGTTTAACTTTTCTCTTAATTCAACATC	This study	5.6 kb
AC-L*cox*1R	TTGCTGAAGTAAAATAAGCTCGTGTATCAA	This study	

### Long-PCR amplification and sequencing

The nucleotide sequences obtained from these three genes were then used to design *A. cerana*-specific primer sets for long PCR reactions. Three overlapping long PCR fragments covering the entire mt genome of *A. cerana* were obtained using the long PCR primers sets (Table 7). The Long-PCR reaction volume amounted 50 µl containing 28.5 µl sterile deionized water, 5.0 µl 10×LA PCR Buffer(Mg^2+^ free), 5.0 µl MgCl_2_ (25 mM), 8.0 µl dNTPs (2.5 mM each), 0.5 µl each primer (50 pmol/µl), 0.5 µl LA *Taq* DNA polymerase (5 U/µl, Takara) and 2 µl DNA template (40 ng/µl). Long-PCR cycling conditions used were initial denaturation step at 92°C for 2 min, followed by 30 cycles of denaturation at 92°C for 10 s, primer annealing at 50°C for 30 s and elongation at 60°C for 10 min during the first 10 cycles, and then an additional 10 s per cycle during the last 20 cycles. The final elongation step was continued at 60°C for 10 min. All amplifications were done on a T-Gradient thermocycler (Biometra, Germany). The three long-PCR fragments were sequenced using a primer-walking strategy. All sequencing was performed with ABI PRISM Big Dye terminator chemistry and analyzed on ABI 3730 automated sequencers (Applied Biosystems, USA).

### Gene annotation and sequence analysis

Sequences were assembled manually and aligned against the complete mt genome sequence of *A. mellifera* using the computer program Clustal×1.83 [55] to identify gene boundaries. The open-reading frames and codon usage profiles of protein-coding genes were analyzed using the program MacVector 4.1.4 (Kodak, version4.0). Translation initiation and translation termination codons were identified based on comparison with the mt genome of *A. mellifera*. The amino acid sequences inferred for the mt genes of the *A. cerana* were aligned with those of *A. mellifera* by using Clustal×1.83. Based on pairwise alignments, amino acid identity (%) was calculated for homologous genes. Codon usage was examined based on the relationships between the nucleotide composition of codon families and amino acid occurrence, where the genetic codons are partitioned into AT rich codons, GC-rich codons and unbiased codons. For analyzing ribosomal RNA genes, putative secondary structures of 22 tRNA genes were identified using tRNAscan-SE [56], of the 22 tRNA genes, 19 were identified using tRNAscan-SE, the other 3 tRNA genes were found by eye inspection, and rRNA genes were identified by comparison with the mt genome of *A. mellifera*.

### Phylogenetic analyses

Phylogenetic relationship among the Hymenoptera were performed using the 14 Hymenoptera species (Table 1) as ingroup, plus the mt DNA sequence of *A. cerana* obtained in the present study, using one Reduviidae species (*Triatoma dimidiata*, GenBank accession number AF301594) as the outgroup, based on amino acid sequences of 13 protein-coding genes. Amino acid sequences for each gene were individually aligned using Clustal×1.83 [55] under default setting, and then concatenated into single alignments for phylogenetic analyses. Three methods, namely neighbor joining (NJ), maximum likelihood (ML) and maximum parsimony (MP), were used for phylogenetic re-constructions. Standard unweighted MP was performed using package Phylip 3.67 [57]. NJ analysis was carried out using PAUP 4.0 Beta 10 programme [58], and ML analysis was performed using PUZZLE 4.1 under the default setting [59]. The consensus tree was obtained after bootstrap analysis, with 1000 replications for NJ and MP trees, and 100 for ML tree, with values above 50% reported.

## Supporting Information

Figure S1 **Inferred secondary structures of 22 tRNAs found in** ***Apis cerana*** **mtDNA.**
(DOC)
