# Supplementary figures and images for: The Complete Mitochondrial Genome of the Asiatic Cavity-Nesting Honeybee Apis cerana (Hymenoptera: Apidae)
Source: PLoS One. 2011 Aug 12;6(8):e23008. doi: 10.1371/journal.pone.0023008 (PMC3155526; doi:10.1371/journal.pone.0023008)

**Figure S1 Inferred secondary structures of 22 tRNAs found in *Apis cerana* mtDNA.**


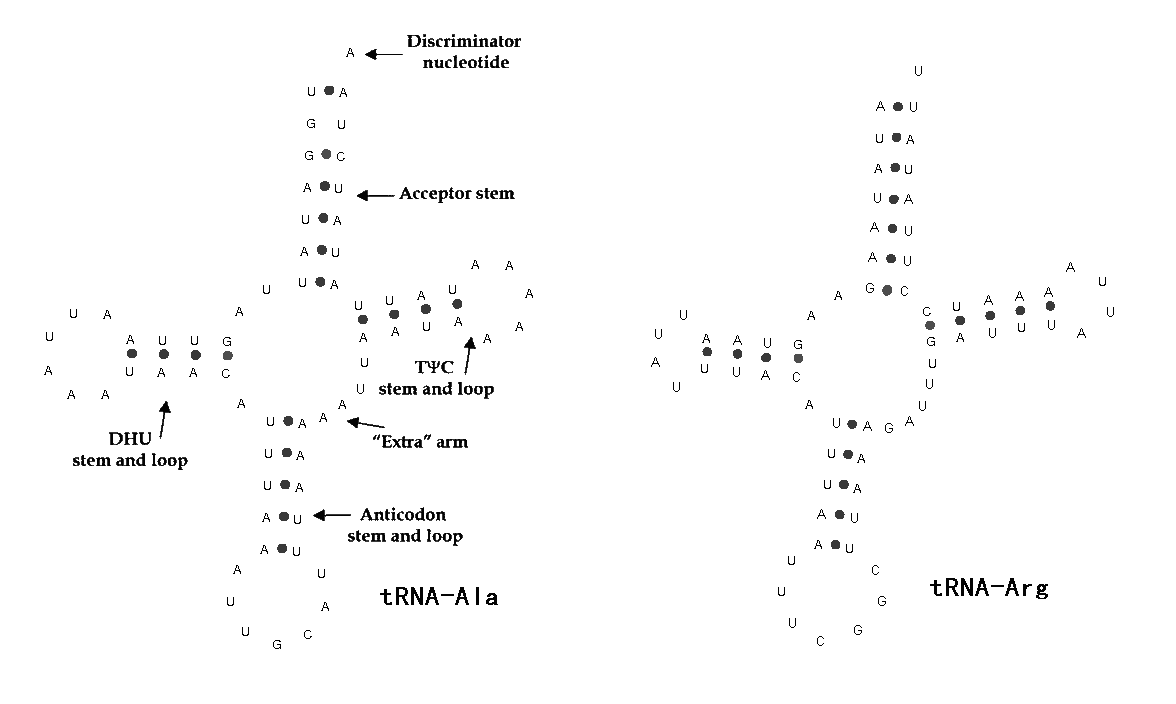

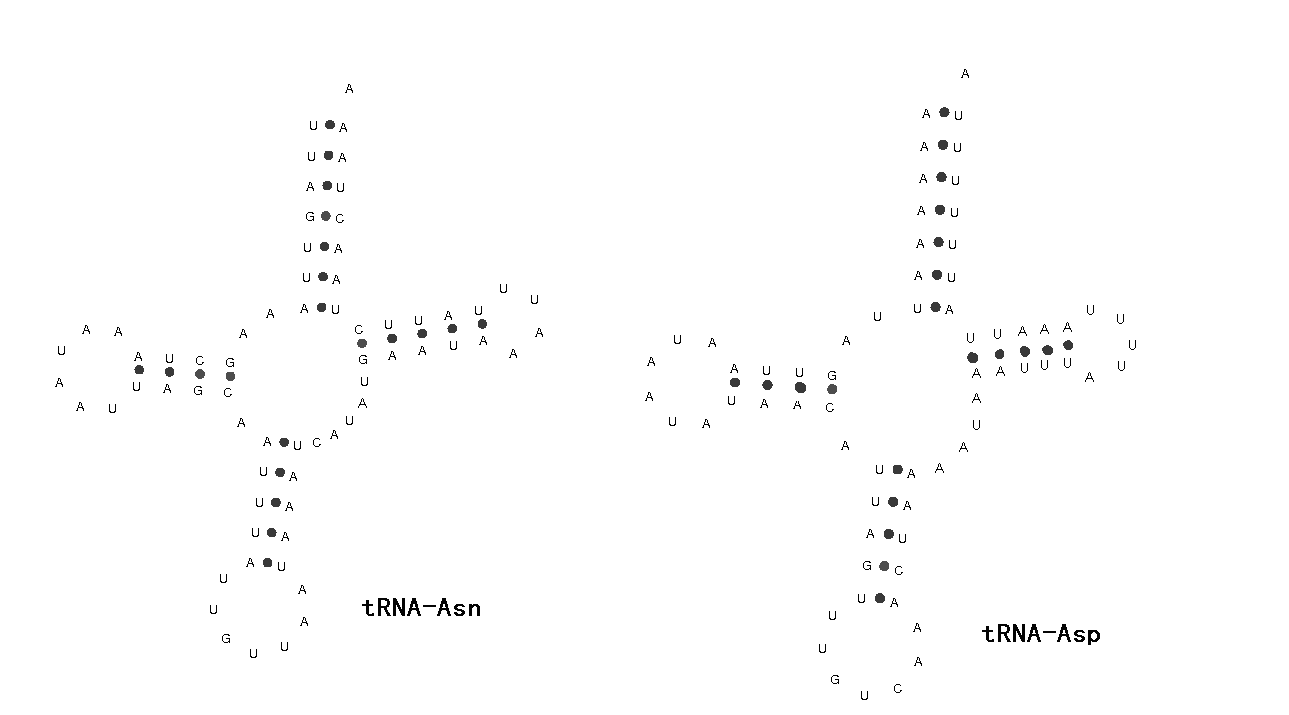


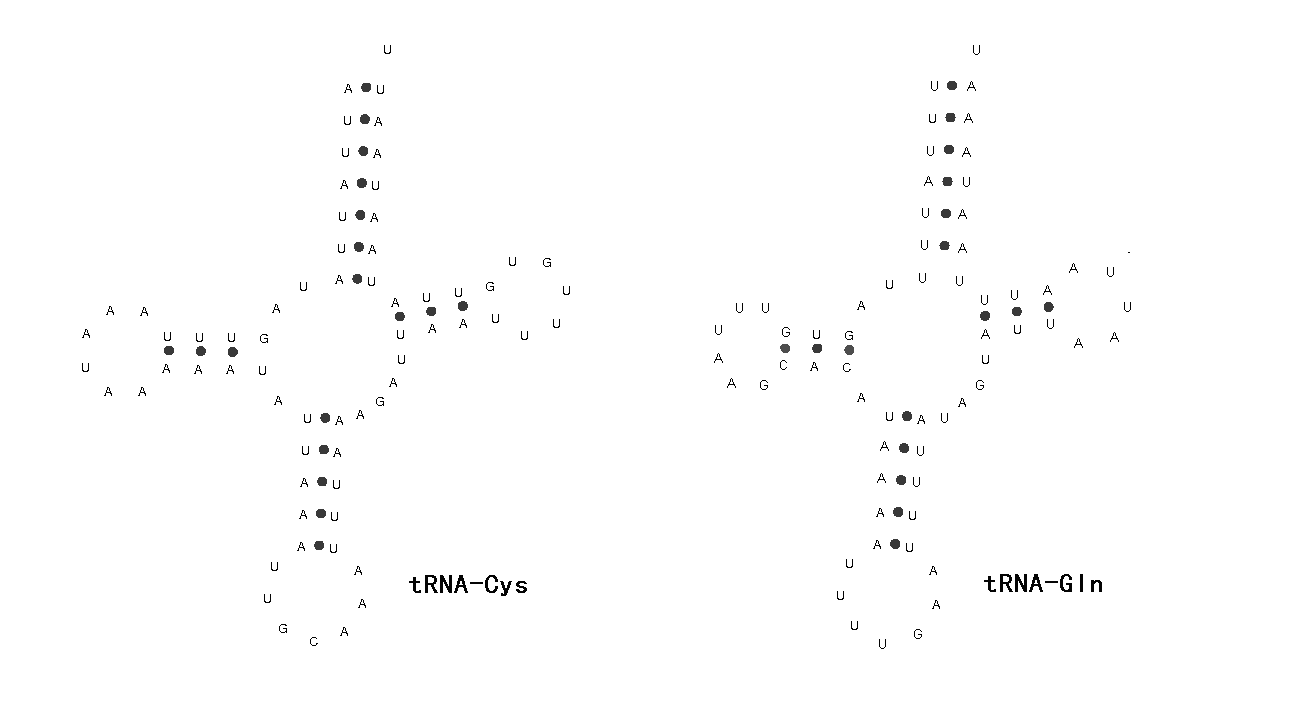

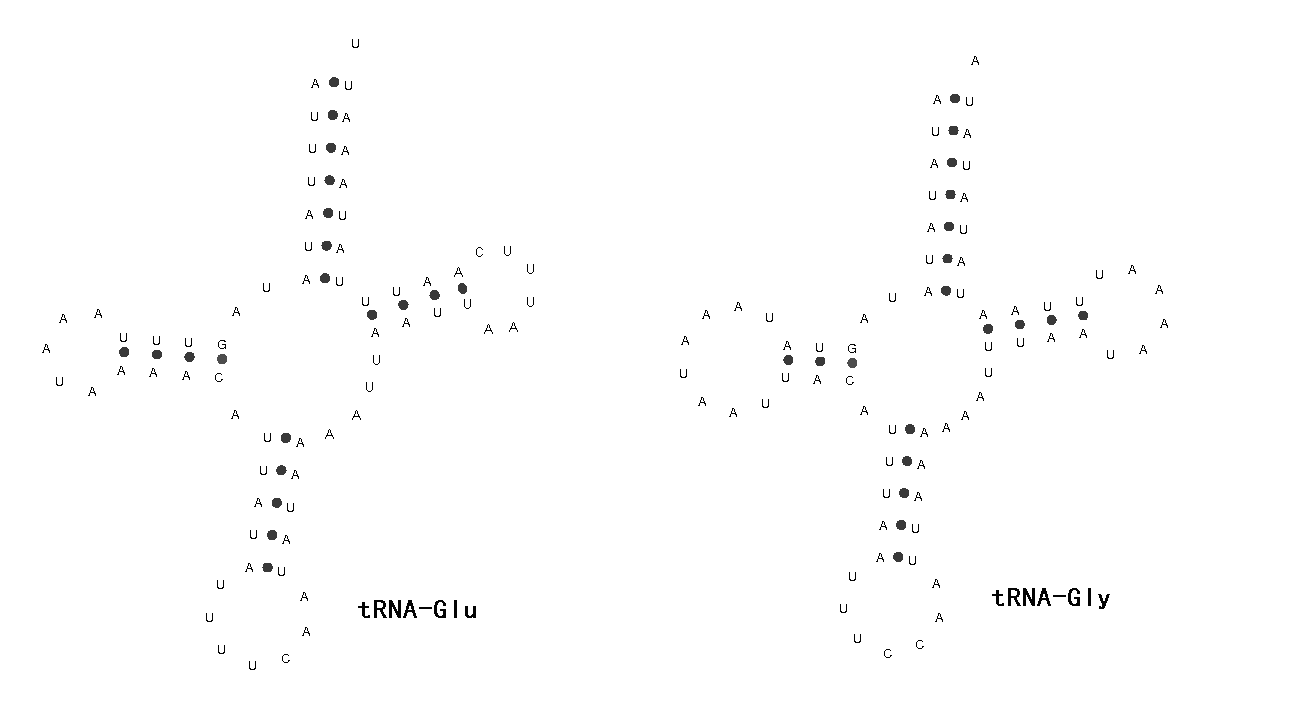


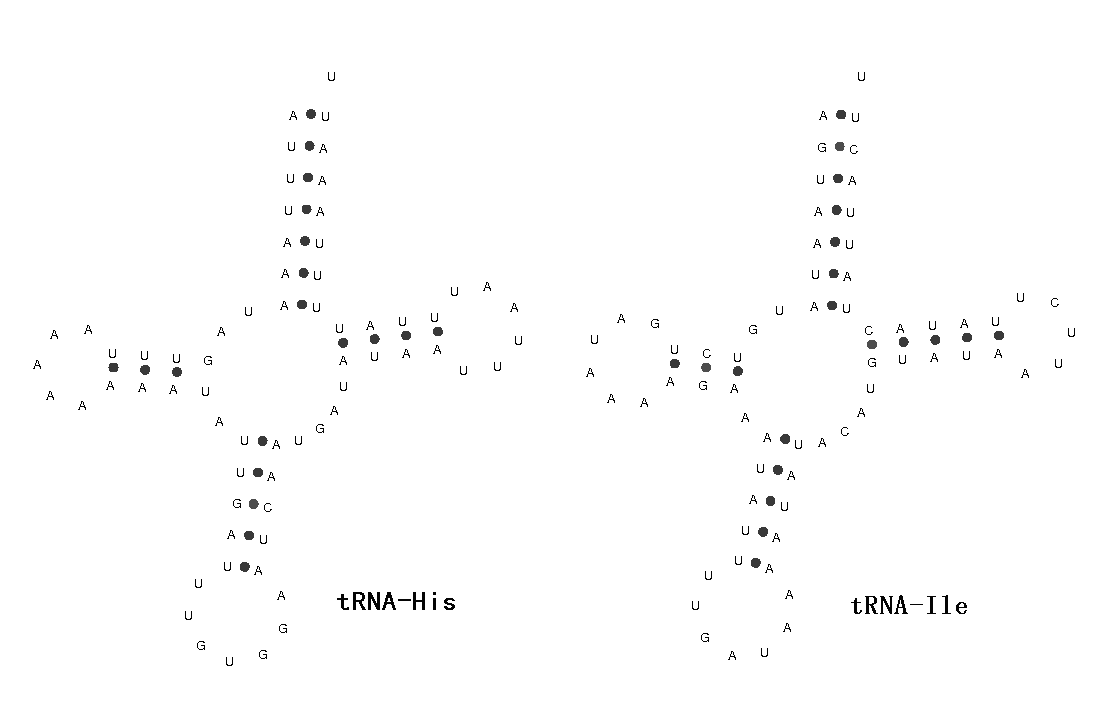

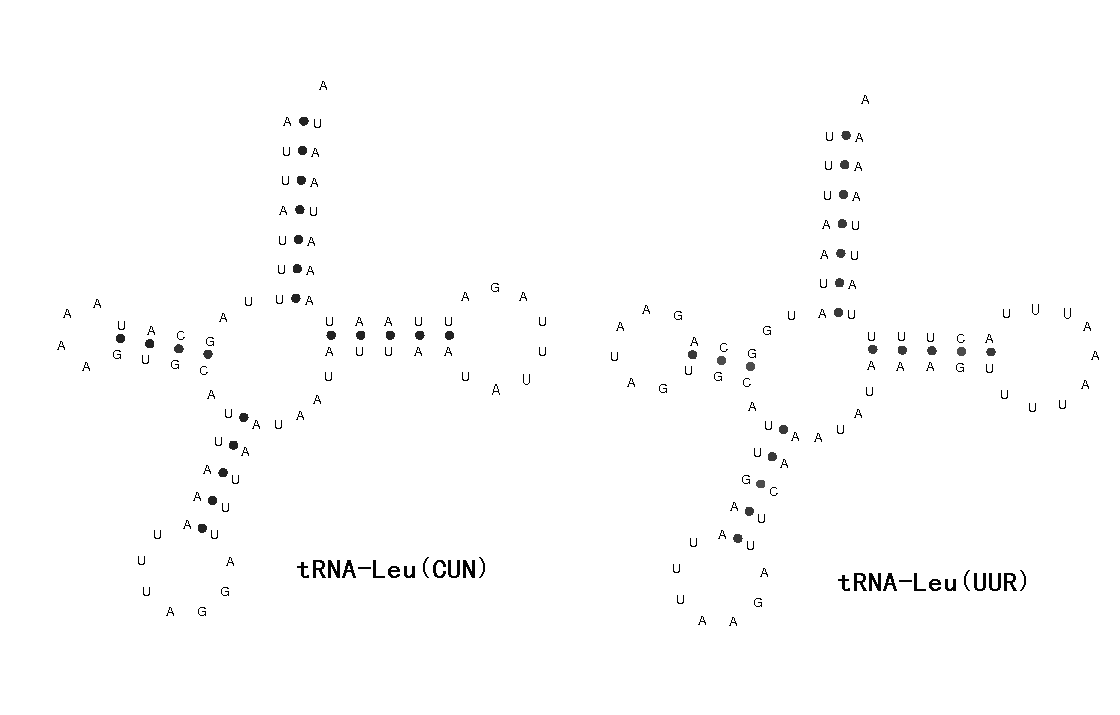


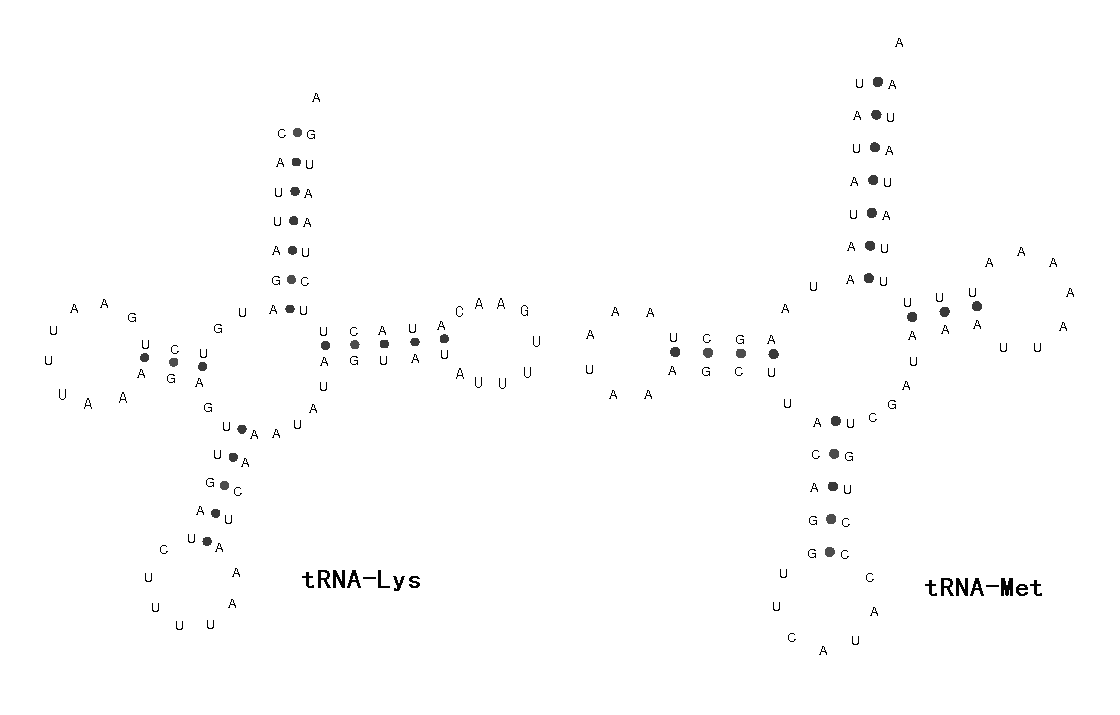

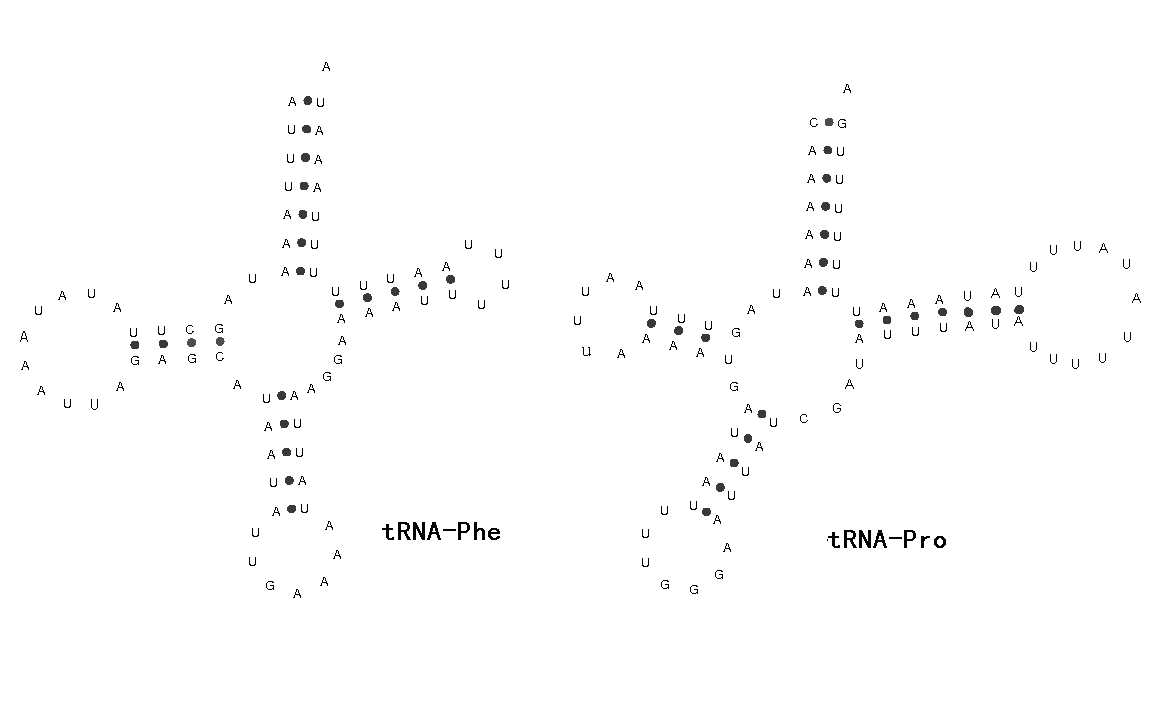


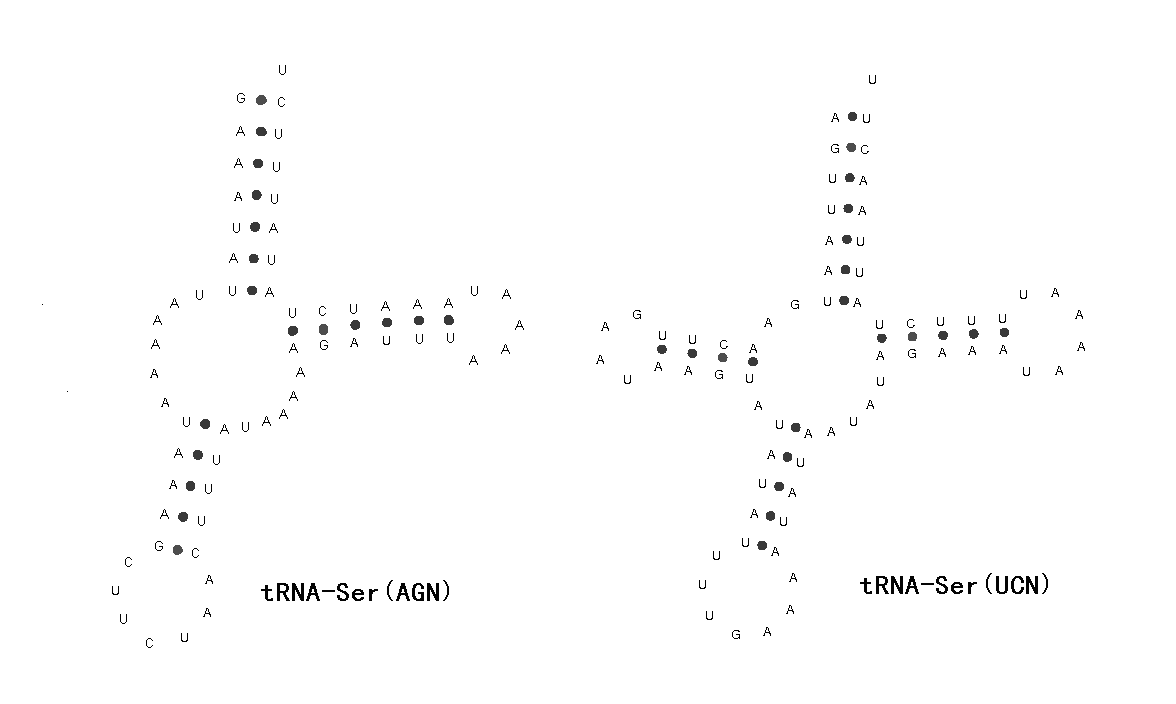

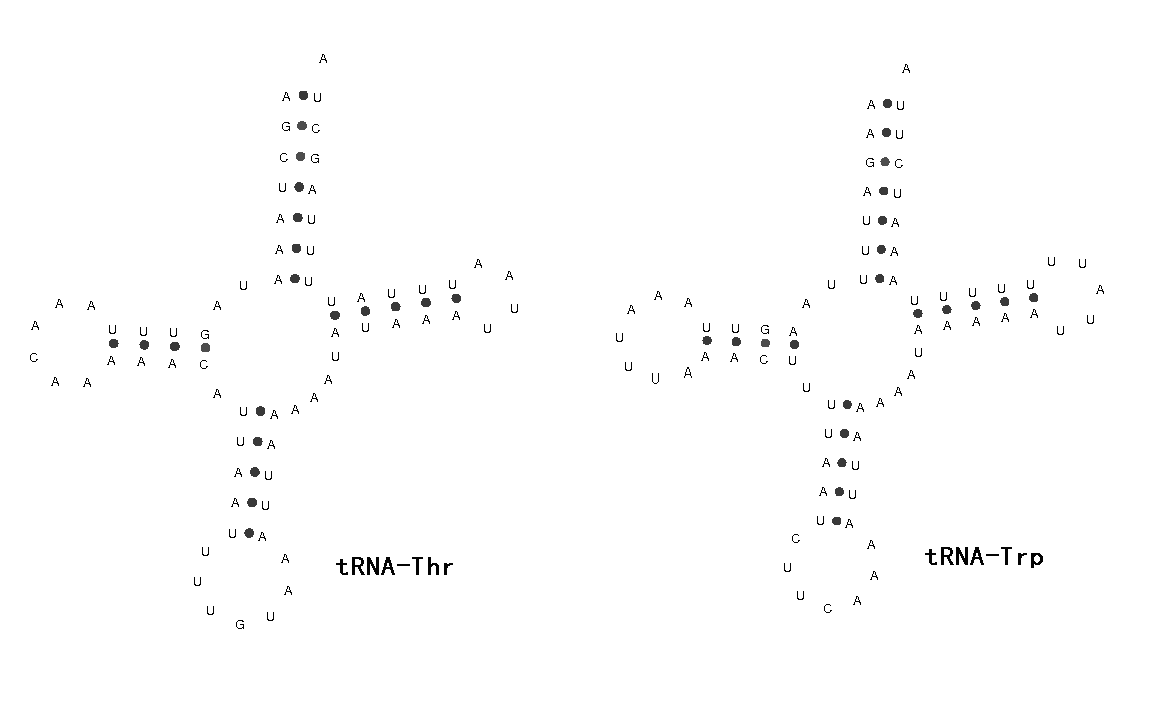


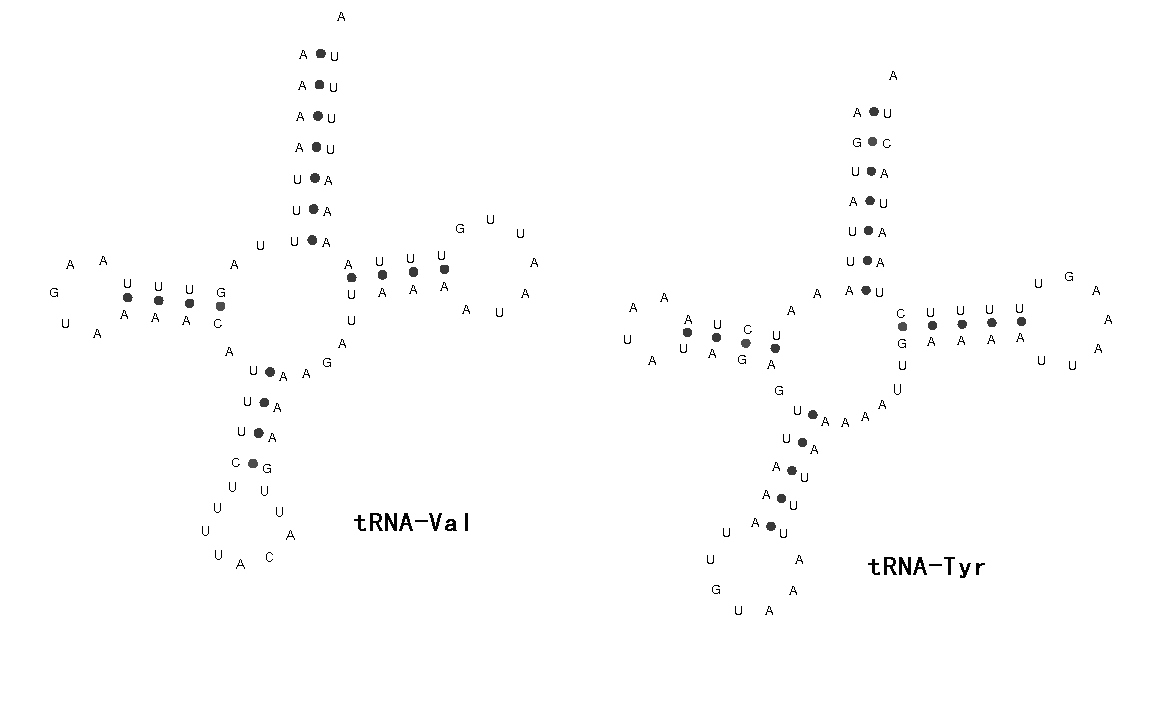

Supplement: Figure S1 — Inferred secondary structures of 22 tRNAs found in Apis cerana mtDNA. (DOC) [file pone.0023008.s001.doc]
